# Overview of the Pathogenesis and Treatment of SARS-CoV-2 for Clinicians: A Comprehensive Literature Review

**DOI:** 10.7759/cureus.10357

**Published:** 2020-09-10

**Authors:** Christian Voto, Paul Berkner, Carol Brenner

**Affiliations:** 1 Internal Medicine, College of Osteopathic Medicine, University of New England, Biddeford, USA; 2 Pediatrics, College of Osteopathic Medicine, University of New England, Biddeford, USA; 3 Obstetrics and Gynecology, College of Osteopathic Medicine, University of New England, Biddeford, USA

**Keywords:** covid19, sars-cov-2, covid induced ards, covid-19 management, sars-cov-2 infection, sars-cov-2 pathogenesis, sars-cov-2 induced ards, sars-cov-2 treatment, remdesivir, dexamethasone

## Abstract

In December 2019, numerous cases of “pneumonia of unknown origin” were presenting throughout Wuhan, China. The pathogen was described to be a novel coronavirus and was subsequently classified as SARS-CoV-2 (severe acute respiratory syndrome coronavirus 2) due to similarities in its pathogenesis and conserved replicase sequence with SARS-CoV-1 (severe acute respiratory syndrome coronavirus 1). Containment measures were initiated; however, the virus began to spread rapidly to countries around the world, and on March 11, 2020, the World Health Organization (WHO) declared a worldwide pandemic. Since the WHO’s declaration, the scientific community has produced an abundance of information about this virus. In this report, we provide a comprehensive review of original articles, clinical trials, and case series in order to produce a concise overview of the pathogenesis and treatment of SARS-CoV-2 (COVID-19 [coronavirus disease 2019]) for clinicians. This review includes data on the roles of the S protein, ACE2 (angiotensin-converting enzyme 2) receptor, and various human secretory proteases, such as transmembrane protease/serine subfamily member 2 and furin, in the pathogenesis of SARS-CoV-2. In addition, a thorough review of treatment options including oxygenation/ventilation strategies, dexamethasone, remdesivir, chloroquine/hydroxychloroquine, immune-based therapies, and anticoagulation are included. Information on this topic is changing rapidly but the authors believe that this review serves as an accurate representation of the current state of knowledge on these topics.

## Introduction and background

For many years, coronaviruses were considered to be inconsequential pathogens causing “the common cold” in immunocompetent people. In fact, coronaviruses account for roughly 10-30% of upper respiratory infections each year [1-2]. However, in more recent years the view on the coronavirus family has changed due to the emergence of severe acute respiratory syndrome coronavirus (SARS-CoV-1) in 2002 and Middle East respiratory syndrome coronavirus (MERS-CoV) in 2012, both of which led to epidemics of considerable morbidity and mortality [1-2].

Throughout December 2019, numerous cases of “pneumonia of unknown origin” were reported throughout Wuhan, China. By January 7, 2020, next-generation sequencing revealed that the pathogen was a novel coronavirus (2019-nCoV), within the B*etacoronavirus genus*. Further analysis revealed that this 2019-nCoV had similarities in its receptor-binding domain (RBD) and conserved replicase sequence with SARS-CoV-1, suggesting that they were in the same subgenera (*Sarbecovirus*) [3-6]. These similarities in structure were used to classify the 2019 n-CoV as SARS-CoV-2.

Containment measures were initiated; however, the virus began to spread rapidly to countries around the world. On January 20, the first case was reported in the United States, and on March 11, 2020, the World Health Organization (WHO) declared a worldwide pandemic [7]. Since then, the virus has affected over 5.7 million patients in the United States and over 23.6 million patients worldwide [8].

Since the WHO’s declaration, the scientific community has produced an abundance of information about this virus. This literature review aims to provide a concise yet comprehensive overview of the basic pathogenesis and treatment of SARS-CoV-2 for clinicians.

## Review

Study acquisition

Articles were searched for in PubMed and Google Scholar. Keywords used were SARS-CoV-2, COVID-19, pathogenesis, SARS-CoV-1, MERS-CoV, S protein, cytokine storm, presentation, risk factors, outcomes, pediatrics, complications, and treatment. In addition, official webpages from the United States, CDC (Centers for Disease Control and Prevention), NIH (National Institutes of Health), WHO, and the International Committee of Taxonomy of Viruses, were included as references. Given the rapidly evolving subject matter of this review, articles in the pre-publication process were taken into consideration. A total of 146 references were collected and analyzed by the authors. Ultimately, 50 of these references were included after the elimination of redundancies and the use of various selection measures, including date of publication and impact factor.

Pathogenesis

Coronaviruses primarily target epithelial cells and are generally associated with respiratory and gastrointestinal infections [2,4]. These viruses attach to target cell receptors and release their genomes into target cells through fusion of the viral envelope with the host plasma membrane [4]. They utilize the spike (S) protein, which is crucial for determining tropism and transmissibility of the virus.

**Figure 1:**
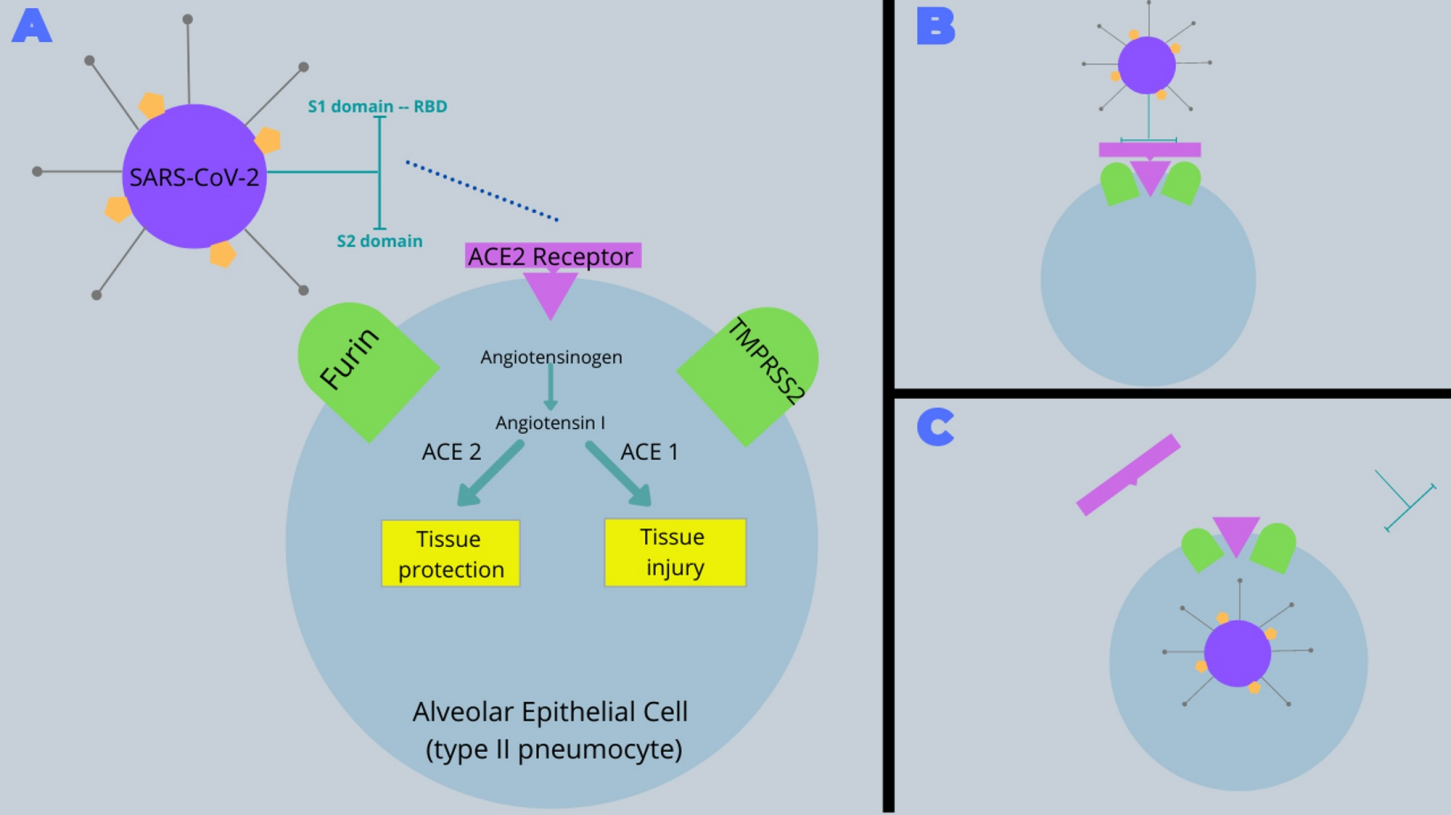
Pathogenesis of SARS-CoV-2 (A) SARS-CoV-2 utilizes a spike (S) protein on its surface in order to bind to ACE2 receptors on the surface of human alveolar epithelial cells. The S protein is divided into two domains, S1 and S2, which are responsible for receptor binding and cell membrane fusion, respectively. The S1 domain contains the RBD, which has the specific ACE2 receptor affinity. (B) Human secretory proteases, such as TMPRSS2 and furin, localize to virally-target cells. (C) These proteases enhance viral entry into host cells through the proteolysis of both the S1, S2, and ACE2 proteins (S1/S2 priming and ACE2 down-regulation). The renin-angiotensin system has a crucial role in acute lung injury and ACE2 serves a protective role against that lung injury. ACE2 downregulation therefore contributes to the severity of illness [2-6,9-14]. ACE2, angiotensin-converting enzyme 2; RBD, receptor-binding domain; TMPRSS2, transmembrane protease/serine subfamily member 2

The S protein is divided into an S1 domain and an S2 domain, which are responsible for receptor binding and cell membrane fusion, respectively [3,5-6]. The S1 domain is what contains the RBD, which has specific receptor affinity. In prior coronavirus outbreaks, SARS-CoV-1 targeted the angiotensin-converting enzyme 2 (ACE2) receptor, whereas MERS-CoV utilized the dipeptidyl peptidase 4 (DPP4) receptor [2,9-10]. When comparing SARS-CoV-1 with MERS-CoV, major differences in their RBDs account for their different receptor specificities [2]. Similarly, SARS-CoV-2 also targets ACE2 receptors on human respiratory epithelial cells, suggesting a similar RBD structure to SARS-CoV-1 [5,11]. The pathogenesis is outlined in Figure 1.

In addition, various human secretory proteases, such as transmembrane protease/serine subfamily member 2 (TMPRSS2) and furin, have been implicated in the pathogenesis of coronaviruses [12-14]. Human secretory proteases are responsible for the cleavage “priming” of various viral surface receptors and adhesion molecules and thereby enhancing viral fusion with host cell membranes. In both SARS-CoV-1 and SARS-CoV-2 infected patients, TMPSSS2 has the ability to localize to viral-targeted cells and enhance entry through the proteolysis of both the S1 and ACE2 proteins, which enhances membrane fusion and viral uptake (S1 priming and ACE2 downregulation) [12-14].

The proteolysis of ACE2 not only promotes viral entry but also seems to be a key mechanism for the severity of lung disease [10]. The renin-angiotensin system has a crucial role in acute lung injury in that the ACE2 receptor has a protective role against acute lung failure [10]. ACE2 is therefore a key negative regulatory factor for severity of lung disease and thus its downregulation contributes to the severity of illness [10].

Like TMPRSS2, furin is also responsible for the cleavage “priming” of SARS-CoV-2 surface receptors. Researchers have identified a furin cleavage site in the S2 domain of SARS-CoV-2, thus suggesting that S2 priming serves a role in the pathogenesis [14]. Interestingly, this furin cleavage site has not been observed among other viruses within its subgenera (i.e. SARS-CoV-1). However, it is present among more genetically-distant coronaviruses, such as MERS-CoV, suggesting a possible convergent evolutionary pathway between the two. Furthermore, the high expression of furin in human lungs may help to explain why SARS-CoV-2 has more efficiently spread throughout the population in comparison to SARS-CoV-1 [14].

Infection leads to increased levels of cytokines (interferon γ, interleukin-1, and interleukin-6), chemokines (CXCL10 and CCL2), and inflammatory markers (C-reactive protein, procalcitonin, and ferritin) [15-17]. This dysregulated host immune response to SARS-CoV-2 lung infection leading to exuberant cytokine release (cytokine storm) and resultant immune-mediated tissue injury has been postulated as a critical pathogenic factor in the progression to acute respiratory distress syndrome (ARDS) and other end-organ complications [15-17].

ARDS is the most common medical complication seen with SARS-CoV-2, and it contributes to a significant amount of the morbidity and mortality associated with the illness. Therefore, it is considered the hallmark immune-mediated clinical consequence [15]. Other complications include shock, acute kidney injury, acute cardiac injury, liver dysfunction, coagulopathy, infection, and even thrombotic disease in otherwise low-risk patients [17-19]. The proportion of patients experiencing each of these complications has been outlined in Table 1. These complications are experienced at higher proportions among severe SARS-CoV-2 cases and therefore serve as major contributors to both intensive care unit (ICU) admission and overall mortality [17-19].

**Table 1:** Complications associated with SARS-CoV-2 The proportion of complications among lab-confirmed SARS-CoV-2 patients compared to the proportion seen among patients labeled with severe SARS-CoV-2 [17-19]. AKI, acute kidney injury; ARDS, acute respiratory distress syndrome; ICU, intensive care unit; SARS-CoV-2, severe acute respiratory syndrome coronavirus 2

	All Lab-Confirmed SARS-CoV-2 Cases		Severe Cases (Higher Risk Patients, ICU Admission)		
	Wang et al. [18]	Guan et al. [17]	Wang et al. [18]	Guan et al. [17]	Xu et al. [19]
	n = 138	n = 1099	n = 36	n = 173	n = 239
Complications					
ARDS	20%	3%	61%	16%	69%
Shock	9%	1%	31%	6%	NA
Acute cardiac injury	7%	<1%	22%	3%	43%
AKI	4%	2%	8%	4%	50%
Liver dysfunction	NA	22%	NA	39%	80%
Coagulopathy	NA	<1%	NA	<1%	63%
Hospital-acquired infection	NA	NA	NA	NA	17%

Interestingly, the pediatric population is at a lower risk of severe complications and more likely to be asymptomatic or have milder symptoms [20-21]. When compared to adults, children reported symptoms such as fever and cough at lower frequencies and were much less likely to have severe/critical disease (5.9% vs. 18.5%) [20-21]. However, when stratified among age groups, 10.6% of children aged <1 year were severe/critical, indicating that although children manifest in a milder form overall, it varies depending on the age group [20]. It is hypothesized that children have these milder pulmonary disease manifestations due to decreased gene expression of ACE2 receptors on respiratory epithelial cells [22].

However, a newly described post-infectious complication of SARS-CoV-2, known as multisystem inflammatory syndrome in children (MIS-C), is being reported. This is a Kawasaki-like syndrome that presents as fever, prominent gastrointestinal upset, rash, conjunctivitis, and neurological findings. In comparison to conventional Kawasaki’s, children with MIS-C are less likely to have coronary involvement [22]. Although causality between SARS-CoV-2 and MIS-C has not been proven, reports of an increased incidence of Kawasaki-like syndrome in areas heavily affected by the pandemic show an epidemiological link [23].

Treatment

Oxygenation and Ventilation Strategies

Currently, there is no strong evidence to suggest that ventilator management of patients suffering from ARDS due to SARS-CoV-2 should differ from standard ARDS management of patients with other viral pneumonias. This strategy includes low tidal volume (4-8 mL/kg of predicted body weight) and high positive end-expiratory pressures (PEEPs) [24]. Due to the poor lung compliance seen with advanced stage SARS-CoV-2 ARDS, patients require a high PEEP in order to improve overall lung recruitment and oxygenation. The panel suggests that the PEEP be individualized for the patient, with the clinician monitoring the patient’s response as well as for signs of hypotension or barotrauma [24].

Interestingly, several studies have found that in contrast to other forms of ARDS, some patients with SARS-CoV-2-induced ARDS may present with normal lung compliance, and thus a high PEEP in these individuals may subject them to barotrauma or reduced cardiac output [25-26]. One study characterized the stages of SARS-CoV-2-induced ARDS as “type L” and “type H”. “Type L” occurs soon after the onset of respiratory distress and the patient’s lungs retain their normal lung compliance, whereas “type H” occurs after progression of the patient’s ARDS, resulting in clinical deterioration and lower lung compliance. Therefore, the study suggests that it may be helpful to adjust the ventilatory approach based on the patients underlying stage and physiology [25]. Additionally, a study examining 17 patients on their second or third day of ICU admission found that a 29% reduction of PEEP resulted in improved lung compliance, reduced hypercapnia, improved fluid balance, and decreased need for vasopressor therapy [26]. However, these findings have not been found among other studies and are thus not generalizable to all SARS-CoV-2 patients; therefore, these strategies cannot be recommended.

Current guidelines recommend that patients on standard oxygen supplementation with refractory hypoxia should be trialed on high-flow nasal cannula (HFNC) over non-invasive positive-pressure ventilatory (NIPPV) strategies, such as bilevel positive airway pressure (BiPAP) and continuous positive airway pressure (CPAP), as long as there is no urgent need for intubation [24,27]. This recommendation stems from the increased risk of aerosol generation and subsequent viral transmission associated with NIPPV. However, if HFNC is not available, then a trial of NIPPV is warranted [27-28].

Prone positioning improves oxygenation and outcomes in intubated patients with ARDS due to improved ventilation-perfusion matching and alveolar recruitment [29]. Awake prone positioning in SARS-CoV-2 patients who require supplemental oxygen therapy is something that is also being explored. Several case series examining its efficacy have found improvements in oxygen saturation and respiratory rates in patients who can adjust their position independently and tolerate lying prone [30-31]. Current guidelines recommend against the use of awake prone positioning to treat refractory hypoxemia in patients who otherwise require immediate intubation [24].

Corticosteroids (Dexamethasone)

Current guidelines recommend the use of dexamethasone (6 mg/day for 10 days) in patients with SARS-CoV-2 who require supplemental oxygen and/or mechanical ventilation; however, it is not currently recommended for patients off supplemental oxygen [24]. SARS-CoV-2 can result in a severe systemic inflammatory response, which can cause lung injury, ARDS, and multisystem organ dysfunction. It is therefore presumed that the potent anti-inflammatory effects of corticosteroid therapy may help to mitigate or prevent these viral complications.

Corticosteroid use in critically-ill patients suffering from ARDS is a topic that has been abundantly covered in the literature [32-34]. Several multi-center randomized control trials have shown that the early introduction of dexamethasone or methylprednisolone to patients with moderate-to-severe ARDS can reduce both mechanical ventilation and overall mortality with comparable adverse effects [32,34]. However, their impact on patient survival varied greatly depending on the duration of ARDS prior to treatment initiation with corticosteroids. For example, patients receiving corticosteroids greater than 14 days after the onset of ARDS saw no benefit in mortality reduction [33]. Despite the strong evidence supporting its use in early ARDS, corticosteroid use for the treatment of viral infections has been a controversial due to concerns for possibly delayed viral clearance and subsequent increase in illness duration and poor outcomes.

Several published retrospective cohort/case series studies as well as one preliminary multicenter randomized control trial (RECOVERY trial) on the use of steroids have been presented. The results from early retrospective studies and case series on corticosteroids have been quite mixed, leading many physicians to question the role of steroid therapy in SARS-CoV-2. One study on 213 patients demonstrated that the early use of methylprednisolone (0.5-1 mg/kg/day for three days) resulted in a reduction in escalation of care, mortality, and mechanical ventilation in patients with moderate-to-severe SARS-CoV-2 pneumonia [35]. While a retrospective review of critically-ill patients from China revealed that patients treated with methylprednisolone (median dosage of 40 mg/day for eight days) were subject to increased risk of multi-organ dysfunction and a potentially increased risk of death [36].

The first multicenter randomized control (RECOVERY) trial was launched in March 2020 in an effort to explore more of the potential benefits of corticosteroids in the treatment of SARS-CoV-2 [37]. The study compared patients treated with standard of care (SOC) plus 6 mg/day of dexamethasone for 10 days to SOC alone. Preliminary analysis of 6,425 patients (2,104 from the dexamethasone arm and 4,321 from the control arm) revealed statistically significant reductions in 28-day mortality among patients within the treatment arm. This effect seems to be influenced by the patient’s baseline SARS-CoV-2 severity. Patients on mechanical ventilation or receiving supplemental oxygenation saw reductions in 28-day mortality, meanwhile patients not on oxygen therapy at enrollment saw no reduction. While this trial serves as the basis for the NIH’s recommendation for the use dexamethasone in SARS-CoV-2 patients who require ventilator or supplementary oxygen support, the results should still be interpreted with caution as they are preliminary [37]. Additionally, review of secondary endpoints, such as need for renal replacement therapy, cardiac arrhythmias, and receipt/duration of ventilation for patients among the treatment arm, is warranted [37].

In the event that dexamethasone is unavailable, it is reasonable to use alternative glucocorticoids, such as prednisone, methylprednisolone, and hydrocortisone. The total daily dose equivalencies to 6 mg/day of dexamethasone for these medications are 40 mg, 32 mg, and 160 mg, respectively. While current guidelines state that using these alternatives are appropriate, it is unclear whether they have the same benefit as dexamethasone [24].

Remdesivir

Current guidelines recommend that remdesivir (five-day course) be prioritized for hospitalized SARS-CoV-2 patients who are on supplemental oxygen but do not require high-flow oxygen, noninvasive ventilation, mechanical ventilation, or extracorporeal membrane oxygenation (ECMO) [24]. Remdesivir is a broad-spectrum antiviral medication that works by targeting viral RNA polymerase and terminating viral transcription. This medication was first trialed back in January on a SARS-CoV-2 patient, who later was noted to recover rather remarkably [7]. Early studies showed high in vitro potency effects, favorable side effect profiles, and improvements in oxygen support among patients receiving compassionate remdesivir [38-39]. However, several large clinical trials were launched by both Gilead (SIMPLE trials) and the NIH (ACTT trial) to evaluate the true efficacy of this medication [40-42].

The NIH’s ACTT trial is a multinational, randomized, double-blind, placebo-controlled trial among hospitalized adult patients with SARS-CoV-2 who had an SpO_2_ of <94%, or required supplemental oxygen, mechanical ventilation, or ECMO [40]. Over 1,000 patients were enrolled, of which half received IV remdesivir (one day of 200 mg followed by nine days of 100 mg). Preliminary results indicated a significant reduction in time to recovery among the treatment arm (11 vs 15 days). Subgroup analysis revealed that this benefit was greatest among hospitalized patients with an oxygen requirement or SpO_2_ < 94%. No benefit was observed among patients with no oxygen requirement or SpO_2_ > 94%, or those receiving mechanical ventilation and ECMO [40]. However, given uncertainties in this study’s ability to detect differences in the subgroups, there is question as to whether remdesivir does confer a benefit among mechanically ventilated patients. Nevertheless, these uncertainties along with supply chain limitations with remdesivir have led toward a prioritization in its use for patients in whom efficacy has been demonstrated and those on supplemental oxygen [24].

The Gilead SIMPLE trials are a group of two multinational, randomized, open-label trials that examine the efficacy of remdesivir (5- and 10-day dosing) in severe SARS-CoV-2 patients (SIMPLE 1) as well as compare the dosing regimens to SOC among patients with moderate SARS-CoV-2 (SIMPLE 2) [41-42]. Published preliminary results from 400 patients enrolled in the SIMPLE 1 trial revealed that there is no significant difference in the time to recovery among patients treated with 5 days versus 10 days of remdesivir [41]. Despite the similar rates with both treatment durations, it is still unclear if critically-ill patients are more likely to benefit from longer therapy duration due to the lack of mechanically ventilated patients in the study [41]. The results from the SIMPLE 2 study are not yet available; however, preliminary reports seem promising (press release. SIMPLE 2 trial by Gilead Sciences. June 1, 2020).

Chloroquine, Hydroxychloroquine, and Azithromycin

Current guidelines recommend against the use of high-dose chloroquine (600 mg BID for 10 days) or hydroxychloroquine plus azithromycin for the treatment of SARS-CoV-2 [24]. Chloroquine and its analog hydroxychloroquine have historically been used to treat malaria and certain autoimmune conditions. However, it has been postulated that they may interfere with viral-cell fusion of SARS-CoV-2 through the increase of endosomal pH. In addition, their immune-modulating effects may control the cytokine storm that occurs in critically ill patients. High in vitro potency has been demonstrated; however, concerns for their safety profile exist, specifically cardiotoxic effects (prolonged QTc) [38]. Additionally, the high doses required to reach sufficient antiviral concentrations along with their concurrent use with azithromycin (another QTc prolonging agent) has further exacerbated these concerns.

Thus far, the safety and efficacy of chloroquine and hydroxychloroquine in SARS-CoV-2 have been evaluated through a number of small clinical trials, case series, and observational studies. Studies on chloroquine have shown minimal efficacy in the treatment of SARS-CoV-2 and increased mortality when taking the high-dose regimen (600 mg BID for 10 days) [42-43]. Studies looking at hydroxychloroquine have been inconclusive, with results varying from worse to better outcomes. One study found that hydroxychloroquine was more likely to be given to patients who were more severely ill at baseline (i.e. older, underlying comorbidities) and therefore could not be determined whether hydroxychloroquine administration resulted in an increased or reduced risk of death/intubation [44]. While others have shown no treatment benefits among patients receiving hydroxychloroquine ± azithromycin compared with patients not receiving hydroxychloroquine or SOC alone [45]. Conversely, a potential increased risk of death or cardiac arrest may be associated with their use [44-45]. Given these safety concerns as well as the lack of robust evidence to support their use, the U.S. Food and Drug Administration (FDA) does not approve the use of hydroxychloroquine and chloroquine for the treatment of SARS-CoV-2 [24].

Immune-Based Therapies

On August 23, 2020, the FDA issued an emergency use authorization (EUA) for the use of convalescent plasma in the treatment of SARS-CoV-2, stating that the “potential benefits outweigh the potential risks” (press release. Gupta S, Gumbrecht J, Fox M. US FDA announces emergency authorization for convalescent plasma to treat Covid-19, August 23, 2020). However, more robust evidence is needed before any statement on its true efficacy can be stated. Current guidelines do not recommend either for or against the use of convalescent plasma in the treatment of SARS-CoV-2 [24]. Convalescent plasma utilizes the principle of passive immunity in that individuals who have generated an antibody response to SARS-CoV-2 may donate their antibodies to individuals in an effort to suppress the viral inflammatory response. Several small case-series have reported clinical efficacy and minimal adverse events; however, the evidence remains inconclusive [46-47]. Results from a randomized clinical trial revealed that there was no clinical or mortality benefit observed among patients with severe or life-threatening SARS-CoV-2 who were treated with convalescent plasma. However, this trial was terminated early and therefore the sample was insufficient to determine subtle clinical differences among the treatment arms [48]. In addition, a safety analysis conducted on 20,000 patients who received convalescent plasma through the National Extended Access Program revealed that although adverse events were uncommon (<1%), they did include transfusion-associated circulatory overload (TACO), transfusion-related acute lung injury (TRALI), allergic reactions, and death [49].

Current guidelines recommend against the use of non-SARS-CoV-2 IV immunoglobulins (IVIG) [24]. There are currently no published studies demonstrating the efficacy of non-SARS-CoV-2 IVIG. The panel has recommended against this therapy given that it is unlikely that the plasma of donors without confirmed SARS-CoV-2 infection contain high titers of SARS-CoV-2 neutralizing antibodies. Additionally, it is unknown if the theoretical immunomodulatory benefits of this therapy outweigh the risks. As for SARS-CoV-2 specific IV immunoglobulins, there is currently no available data on its use in treatment, and therefore there is not a recommendation for or against its use [24].

Lastly, the use of interleukin-1 and interleukin-6 inhibitors has also been proposed; however, there are insufficient clinical data available to support their use [24]. Interferons are not recommended due to their safety concerns and limited benefit in other coronavirus infections [24].

Anticoagulation

The incidence of thrombotic disease in individuals affected by SARS-CoV-2 is reported as high as 31% [50]. The precise mechanism behind this pro-coagulable state associated with SARS-CoV-2 is poorly understood. Current guidelines suggest that hospitalized adults with SARS-CoV-2 should receive the same prophylaxis against arterial thrombosis or venous thromboembolism as per the SOC of other hospitalized individuals. Patients with SARS-CoV-2 who do experience a thrombotic evident should be managed with anticoagulation therapy as per SOC as well [24]. Despite the link between SARS-CoV-2 and thromboembolic events, little evidence demonstrating the benefits of using therapeutic dosages of anticoagulants in patients with SARS-CoV-2 exists. One retrospective analysis of 2,773 patients treated either with or without systemic anticoagulation found no difference in overall mortality among the two groups; however, subgroup analysis of ventilated patients did show potential mortality benefit [50]. Nevertheless, there are significant limitations of this study, justifying a need for future prospective clinical trials.

Concomitant Therapies

Patients receiving chronic anticoagulation/platelet therapies should continue these medications after a SARS-CoV-2 diagnosis. In addition, patients who have been prescribed medications such as ACE inhibitors, angiotensin receptor blockers, and HMG-CoA reductase inhibitors for certain chronic conditions prior to a diagnosis of SARS-CoV-2 should not discontinue their therapy [24]. Patients also taking either chronic oral or inhaled steroids should also continue with therapy with possible consideration for stress dosage [24].

## Conclusions

This review provides a concise summary of relevant literature on the pathogenesis and treatment of SARS-CoV-2. Information on this topic is changing rapidly, but the authors believe that this review serves as an accurate representation of the current state of knowledge on these topics. Patients with persistent hypoxemic respiratory failure on conventional oxygen supplementation should be treated with HFNC over NIPPV. Both types of NIPPV (CPAP and BiPAP) are acceptable strategies to treat SARS-CoV-2 for short-term life-threatening respiratory conditions if HFNC is unavailable and there is no urgent need for intubation. If required, endotracheal intubation should be administered by health care providers with extensive airway management experience. Both dexamethasone and remdesivir have demonstrated positive early results in clinical trials. Dexamethasone should be trialed in patients who require supplemental oxygen and/or mechanical ventilation; however, it is not currently recommended for patients off supplemental oxygen. Additionally, remdesivir should be prioritized for hospitalized SARS-CoV-2 patients who are on supplemental oxygen but do not require high-flow oxygen, noninvasive, or invasive ventilation. Dexamethasone seems to have a greater benefit in patients with more severe pulmonary disease (in need of supplemental oxygen), whereas remdesivir seems to help stop the virus from replicating; however, it doesn’t seem as beneficial for some severely ill patients who are suffering from massive inflammation. Thus, the medical community is racing to find the perfect treatment combination against SARS-CoV-2 by blending remdesivir with other drugs to reduce inflammation. Lastly, it has been proposed that convalescent plasma may be beneficial for SARS-CoV-2, and studies are currently underway to evaluate its use for both treatment and prophylaxis of certain high-risk patients. Despite the recent EUA, a lot is still unknown and larger studies are needed.
